# Role of type 2 ryanodine receptor stabilisation in autoimmune cell modulation

**DOI:** 10.1186/s42358-024-00426-3

**Published:** 2024-11-22

**Authors:** Takashi Nawata, Takeshi Honda, Akihiko Sakamoto, Hitoshi Uchinoumi, Takeshi Suetomi, Yoshihide Nakamura, Shunya Tsuji, Hiroki Sakai, Shigeki Kobayashi, Takeshi Yamamoto, Masataka Asagiri, Motoaki Sano, Masafumi Yano

**Affiliations:** 1 Department of Medicine and Clinical Science, Yamaguchi University Graduate School of Medicine, 1-1-1 Minamikogushi, Ube, 755-8505 Japan; 2 Department of Pharmacology, Yamaguchi University Graduate School of Medicine, 1-1-1 Minamikogushi, Ube, 755-8505 Japan; 3 Faculty of Health Sciences, Yamaguchi University Graduate School of Medicine, 1-1-1 Minamikogushi, Ube, 755-8505 Japan

Ryanodine receptors (RyRs) are intracellular Ca^2+^-release channels located in the sarcoplasmic/endoplasmic reticulum membrane, which assume a crucial role in facilitating the deliverance of intracellular Ca^2+^ [1]. A recent investigation by Wang et al. involving type 2 RyR (RyR2)-knockdown experiments reported that RyR2-depleted conventional T cells exhibit regulatory T cell-like suppressive functions in multiple inflammatory or autoimmune contexts [2]. Nonetheless, RyR2 knockdown in patients can be challenging in clinical practice. Therefore, this study aimed to propose RyR2 stabilisation as an alternative strategy for the acquisition of a comparable effect.

Prior research has reported that Ca^2+^ leakage via RyR2 is primarily driven by a reduction in RyR2-calmodulin binding. Based on these findings, we created V3599K-RyR2 knock-in (RyR2^V3599K^/RyR2^V3599K^) mice by replacing the endogenous gene with a gene in which the Valine at 3599th amino acid in the calmodulin binding domain on RyR2 was replaced with Lysine. These mice have indeed a higher affinity for calmodulin, an important modifying protein for RyR2 [3, 4], stabilizing RyR2 and preventing Ca ^2+^ leakage from RyR2. RyR2^V3599K^/RyR2^V3599K^ mice show inhibition of Ca^2+^ leakage via enhanced RyR2-calmodulin binding, which is associated with improvements in RyR2-related disorders, such as catecholaminergic polymorphic ventricular tachycardia and Alzheimer’s disease [4, 5]. These findings provide evidence to indicate the genetic stabilisation of RyR2.

Regarding autoimmune disorders and immune-mediated inflammatory arthropathies, we have reported that treatment with dantrolene, an RyR stabiliser, contributes to a substantial reduction in serum anti-type II collagen immunoglobulin G (IgG) levels in mice with collagen-induced arthritis (CIA), thereby preventing CIA [6]. Furthermore, we and others have reported that dantrolene is a candidate immunomodulator for treating autoimmune disorders and immune-mediated inflammatory arthropathies, including rheumatoid arthritis and multiple sclerosis [6–8].

In addition to the aforementioned findings, we recently established that RyR2 stabilisation assumes a crucial role in suppressing autoantibody induction via antigen sensitisation. Summarily, 8–10-week-old female wild-type *C57BL/6* and RyR2^V3599K^/RyR2^V3599K^ mice were initially immunised with a subcutaneous injection of 100 µg ovalbumin (OVA) emulsified with complete Freund’s adjuvant; an emulsion comprising Freund’s incomplete adjuvant and 100 µg of OVA was administered on day 21 to achieve second immunisation. Serum anti-OVA IgG levels were measured 63 days after the initial immunisation using an enzyme-linked immunosorbent assay kit (Chondrex, Woodinville, WA, USA; cat. no. 3011).

OVA-immunised RyR2^V3599K^/RyR2^V3599K^ mice exhibited a marked reduction in serum anti-OVA IgG levels (Fig. 1), suggesting that RyR2 stabilisation may help to ameliorate autoimmune disorders and immune-mediated inflammatory arthropathies.

**Fig. 1:**
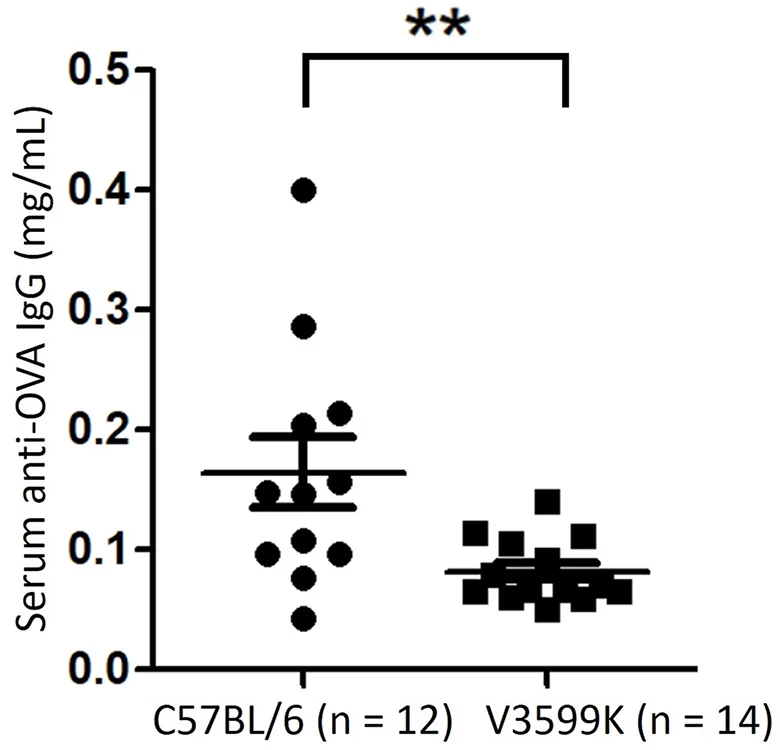
Serum anti-ovalbumin immunoglobulin G levels on day 63 following an initial immunisation. Data were collected from two independent experiments, with values being presented as the means ± standard error of the mean (SEM). A Mann–Whitney U test was performed to compare differences between two independent groups. **p < 0.01; n, number of animals in a group; IgG, immunoglobulin G; OVA, ovalbumin; V3599K, RyR2^V3599K^/RyR2^V3599K^ mice

Conclusively, our findings and the report by Wang et al. [2] suggest that RyR2 stabilisation may augment the suppressive effect on abnormal immune cells, including pathological T cells. Although the association between RyR2 and immune cells warrants further elucidation, we posit that RyR2 stabilisation may be effective in autoimmune cell modulation.

## Data Availability

All data generated and/or analysed during this study are presented in this article.

## Abbreviations

CIA: Collagen-induced arthritis
IgG: Immunoglobulin G
OVA: Ovalbumin
RyR2: Type 2 ryanodine receptor
RyR: Ryanodine receptor
RyR2^V3599K^/RyR2^V3599K^: V3599K- type 2 ryanodine receptor knock-in
